# Blood pressure measurement and blood pressure control in Veterans Affairs medical centers

**DOI:** 10.1111/jch.14684

**Published:** 2023-06-21

**Authors:** Meghan O’ Halloran, Ashley M Hughes, Zhiping Huo, Frances Weaver, Kevin Stroupe, Elizabeth Tarlov, Holly Kramer

**Affiliations:** ^1^ Medicine Service Line Hines Illinois USA; ^2^ Center for Innovation in Complex Chronic Healthcare Edward Hines Jr. Veterans Affairs Hospital Hines Illinois USA; ^3^ Department of Biomedical and Health Information Sciences University of Illinois Chicago Chicago Illinois USA; ^4^ National Institute of Nursing Research National Institutes of Health Bethesda Maryland USA; ^5^ Departments of Public Health Sciences and Medicine Loyola University Chicago Maywood Illinois USA

**Keywords:** antihypertensive therapy, clinical management of high blood pressure (HBP), epidemiology, hypertension in the elderly, hypertension‐general

## Abstract

The Veterans Affairs (VA) medical centers provide care for millions of Veterans at high risk of cardiovascular disease and accurate BP measurement in this population is vital for optimal BP control. Few studies have examined terminal digit preference (TDP), a marker of BP measurement bias, clinician perceptions of BP measurement, and BP control in VA medical centers. This mixed methods study examined BP measurements from Veterans aged 18 to 85 years with hypertension and a primary care visit within 8 VA medical centers. TDP for all clinic BP measurements was examined using a goodness of fit test assuming 10% frequency for each digit. Interviews were also conducted with clinicians from 3 VA medical centers to assess perceptions of BP measurement. The mean age of the 98,433 Veterans (93% male) was 68.5 years (SD 12.7). BP was controlled (<140/90 mmHg) in 76.5% and control rates ranged from 72.2% to 81.0% across the 8 VA medical centers. Frequency of terminal digits 0 through 9 differed significantly from 10% for both SBP and DBP within each center (*P* < .001) but level of TDP differed by center. The highest BP control rates were noted in centers with highest TDP for digits 0 and 8 for both SBP and DBP. Clinicians reported use of semi‐automated oscillometric devices for clinic BP measurement, but elevated BP readings were often confirmed by auscultatory methods. Significant TDP exists for BP measurement in VA medical centers, which reflects continued use of auscultatory methods.

## 1 INTRODUCTION & BACKGROUND

Blood pressure (BP) control remains the most effective intervention for primary and secondary cardiovascular disease (CVD) prevention.^1^, ^2^, ^3^ Hypertension affects over half of Veterans aged 50 years and older,^4^ and CVD prevalence is higher among Veterans compared to the general population^5^ and continues to be a major reason for disability and hospitalization.^6^, ^7^ Two decades ago, the Veterans Affairs (VA) medical centers, which provides care for millions of Veterans across the U.S., implemented system wide strategies to improve the diagnosis and treatment of hypertension. Interventions to improve BP control included use of BP control as a performance measure, automated provider notification of uncontrolled BP, electronic clinical reminders to address hypertension, and systematic return to clinic schedules.^8^ In 2012, Fletcher et al reported that these system wide interventions led to a 3% annual increase in BP control (<140/90 mmHg) across VA medical centers during years 2000−2010.^8^ During this time‐period, BP was measured with the auscultatory method using aneroid sphygmomanometers.

Knowledge regarding optimal BP measurement techniques and hypertension treatment have evolved over the past decade. Hypertension guidelines^1^, ^9^ and a scientific statement from the American Heart Association^10^ now recommends the oscillatory method using automated or semi‐automated devices for BP measurement because this method eliminates terminal digit preference (TDP), the tendency to record a specific digit (e.g., 0) more frequently than others when recording a BP measurement. In addition, automated and semi‐automated devices do not require frequent calibration like aneroid sphygmomanometer devices.^11^, ^12^, ^13^

We recently reported the presence of strong TDP for BP measurements in Veterans receiving care from a single VA patient aligned care team (PACT) and TDP was eliminated with implementation of a standardized BP measurement protocol using semi‐automated oscillometric devices.^14^ The objective of this study was to assess presence of TDP in hypertensive Veterans receiving care from all VA PACT clinics across eight Midwest VA medical centers. This study also determined BP control and explored knowledge and perceptions of BP measurement based on the tenets of the Measure accurately, Act rapidly, and Partner with patients (MAP)^15^, ^16^, ^17^, ^18^ hypertension quality improvement model, a quality improvement program to improve BP control developed by the American Medical Association.

## 2 METHODS

This mixed methods study first examined the presence and extent of TDP using all BP values measured during PACT visits at eight Midwest region VA medical centers. The prevalence of BP control defined as <140/90 mmHg and as <130/80 mmHg was also determined based on the last PACT clinic recorded BP during the study period. We also interviewed PACT clinicians at 3 of the 8 VA medical centers to determine perceptions of BP measurement protocols. The study protocol was approved by the Edward Hines, Jr. VA Institutional Review Board.

### 2.1 Quantitative analyses

#### 2.1.1 Study population

**FIGURE 1:**
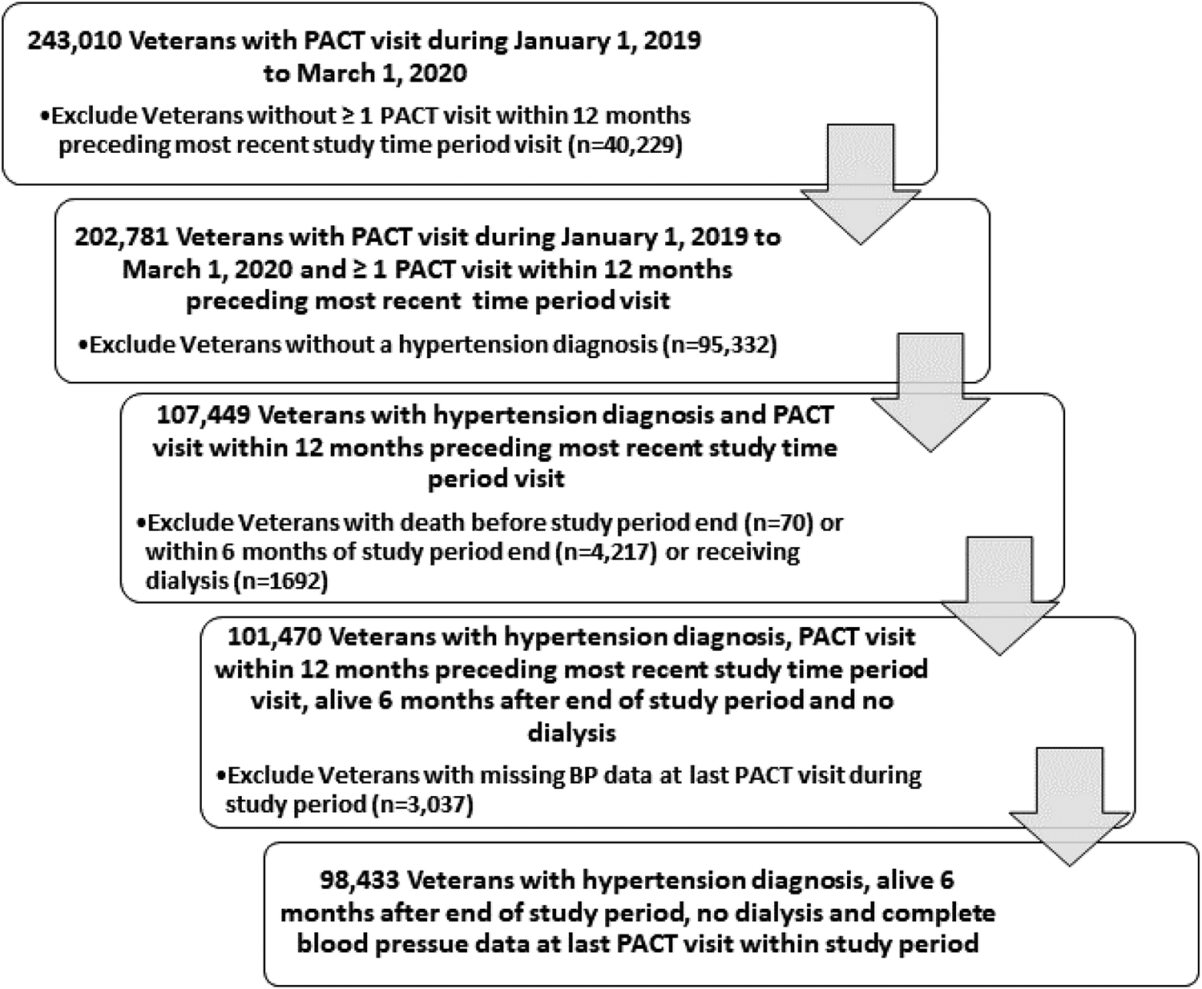
Flowchart of study population for quantitative analysis of blood pressure terminal digit preference and blood pressure control.

Data were obtained from the VA's national Corporate Data Warehouse (CDW), which includes vital signs, patient demographics, outpatient encounter diagnosis, procedure codes (ICD10 and CPT codes), laboratory values, and co‐morbidities. Data from the CDW were extracted for Veterans ages 18–85 years old followed in a PACT clinic affiliated with the 8 medical centers in the VA Great Lakes Health Care System. These 8 VA medical centers include 41 outpatient clinics that provide health care services to almost 800,000 Veterans who reside within the four‐state region (Illinois, Michigan Upper Peninsula, Wisconsin, and Northwest Indiana). A total of 243,010 unique Veterans with at least one PACT clinic visit between January 1, 2019, and March 1, 2020 were identified and 202,781 had at least one PACT clinic visit within 12 months preceding the index visit (see Figure 1). Within this group, over half (*n* = 107,449) had an ICD‐10 diagnosis code of hypertension or their electronic medical record (EMR) problem list included hypertension (Figure 1). Veterans who died before or within 6 months after the index date (*n* = 4287), were receiving dialysis (*n* = 1692), or had no recorded BP value on the index date (*n* = 3037) were excluded. A total of 98,443 Veterans with hypertension and at least one PACT clinic visit with complete BP data during the study period were included in the analysis (Figure 1).

Demographics and co‐morbidity data were obtained from the CDW. Race and ethnicity were self‐reported and presence of diabetes and CVD (arrhythmias, coronary artery disease, heart failure, valvular heart disease, ischemic or hemorrhagic stroke, peripheral arterial disease, carotid artery disease or aortic aneurysm) were based on ICD‐10 diagnosis codes or these diagnoses were included in the EMR problem list. Chronic kidney disease (CKD) was based on an ICD10 CKD diagnosis code, inclusion of CKD in the EMR problem list, or an estimated glomerular filtration rate <60 mL/min/1.73 m^2^ at two time points separated by 90 days prior to the end of the study period.

#### 2.1.2 Blood pressure control

We defined BP control as <140/90 mmHg based on BP recorded during the last PACT clinic visit during the study period (January 1, 2019 through March 1, 2020). We also examined BP control defined as <130/80 mmHg among Veterans with high CVD risk defined as age ≥65 years or presence of diabetes, CKD or CVD.

### 2.2 Statistical analysis

Characteristics of the 98,433 Veterans were compared by BP control defined as <140/90 mmHg. Continuous variables were compared using an unpaired t‐test and categorical variables were compared using a Chi‐square test. Presence of TDP for systolic BP (SBP) and diastolic BP (DBP) was determined for all clinic BP measurements. We also examined TDP for BP measurements ≥140/90 mmHg and <140/90 mmHg, and by medical center. In the absence of TDP, digits 0 through 9 each have a 10% likelihood of frequency. To assess presence of TDP, we used a goodness of fit test to assess the hypothesis of 10% frequency of each digit. All statistical analyses were completed using STATA v 14.0. Statistical significance was set at *p* < .05.

### 2.3 Qualitative analyses

PACT frontline clinical (licensed practical nurses [LPN], registered nurse manager [RN], and primary care physicians [PCP]) working in three of the eight VA medical center PACT clinics provided cognitive interviews framed around the tenets of the hypertension quality improvement model Measure accurately, Act rapidly, and Partner with patients (MAP), a quality improvement program to improve BP control.^15^, ^16^, ^17^ The interviewees worked directly with BP measurement and/or hypertension management within a PACT.

Clinician interview guides were designed to gather frontline clinical staff perceptions and actual practices of BP measurement. Initial questions were developed through multidisciplinary and iterative discussions between hypertension specialists, primary care nurses, and physicians and then revised to elicit components of workflow related to the MAP program.^15^, ^16^, ^17^ Trained research assistants contacted eligible clinic staff and invited staff to participate in an audio‐only interview. Due to IRB restrictions on the scope of research, interviews were limited to up to five clinic staff or clinicians for three VA medical centers and all interviewees provided verbal consent.

### 2.4 Data collection

Each interview was attended by at least two trained members of the study team (AMH, MO, IK). One research team member led the interview with the participant while the other documented notes. The interviewers reviewed field notes scrubbed of identifying information for completeness and accuracy. Two members of the research team (AMH, HK) then reviewed each transcript independently using deductive thematic analysis (DTA)^19^, ^20^ to identify and extract emergent themes related to BP measurement according to the MAP framework.^15^, ^16^, ^17^, ^18^ Coders were in high agreement (99.9%, *Κ* = 0.98) and discrepancies were resolved via consensus.

## 3 RESULTS

### 3.1 Quantitative analyses

The mean age of the 98,433 Veterans with a hypertension diagnosis and PACT clinic visits at the 8 centers in this predominantly male (93.9%) cohort was 68.5 years (SD 12.7). Mean SBP and DBP were 130.8 mmHg (SD 16.1) and 74.8 mmHg (SD 10.6), respectively (Table 1). Veterans self‐reported race as African American in 20.2%, White in 78.0%, Asian in 0.4%, and Alaskan Indian or Pacific Islander in 1.4%. Hispanic ethnicity was reported by 2.7%. The median number of PACT visits was 4.9 and co‐morbidities included CKD in 12.6%, diabetes mellitus in 34.4%, and CVD in 30.4%. BP was controlled (<140/90 mmHg) in 76.5% (*n* = 75, 281) overall and ranged from 72.2% to 81.0% across the 8 centers (Table 2). Among the 75, 301 (76.5%) Veterans with high CVD risk, BP was controlled to <130/80 mmHg in 37.6% ranging from 33.6% to 45.5% across the eight centers (Table 2).

**TABLE 1:** Baseline characteristics of the Veterans with hypertension and VISN 12 PACT visits by blood pressure control based on the last clinic visit.

	Veterans with hypertension *n* = 98, 433	Controlled BP (<140/90 mmHg at last visit) *n* = 75, 281	Uncontrolled BP (≥140/90 mmHg at last visit) *n* = 23, 152	*P*‐value
Mean age, years	68.5 (12.7)	68.5 (12.7)	68.6 (12.8)	.1
Age group				
18‐65 years, %	32.0	31.6	33.3	<.001
65‐85 years, %	68.0	68.4	66.7	<.001
Male, %	93.9	93.8	94.1	.06
Race				<.001
Black/African American, %	20.2	18.9	24.5	
White, %	78.0	79.3	73.8	
Asian, %	0.4	0.4	0.3	
Alaskan Indian/Pacific Islander	1.4	1.5	1.4	
Hispanic Ethnicity, %	2.7	2.7	2.6	.5
Mean SBP mmHg	130.8 (16.1)	124.4 (10.8)	151.6 (12.8)	<.001
Mean DBP mmHg	74.8 (10.6)	72.4 (9.1)	82.8 (11.5)	<.001
Diabetes mellitus, %	34.4	34.9	33.0	<.001
Chronic kidney disease, %	12.6	12.7	12.3	.1
Cardiovascular disease, %	30.1	31.5	25.4	<.001
^a^# Clinic visits	4.9	4.9	4.7	<.001

^a^Data shown as median.

**TABLE 2:** Blood pressure control among Veterans with diagnosed hypertension by site.

VA Center	Total N	% (N) BP Control (<140/90 mmHg)	^a^Number with High CVD risk	% (N) BP Control (<130/80 mmHg) with High CVD risk
A	15729	73.9 (11,618)	14, 762	33.6 (4958)
B	10284	77.7 (7986)	9,222	39.2 (3613)
C	7231	79.6 (5754)	6,624	41.0 (2716)
D	19019	81.0 (15403)	18,235	45.5 (8294)
E	8237	72.2 (5943)	7,040	30.7 (2160)
F	11561	74.4 (8605)	10,360	34.8 (3607)
G	8376	80.6 (6755)	7,123	38.2 (2772)
H	17996	73.4 (13217)	16,171	34.1 (5514)
Total	98433	76.5 (75281)	89,537	37.5 (33,584)

^a^High CVD risk defined as age ≥65 years and/or presence of cardiovascular disease, chronic kidney disease or diabetes mellitus.

**FIGURE 2:**
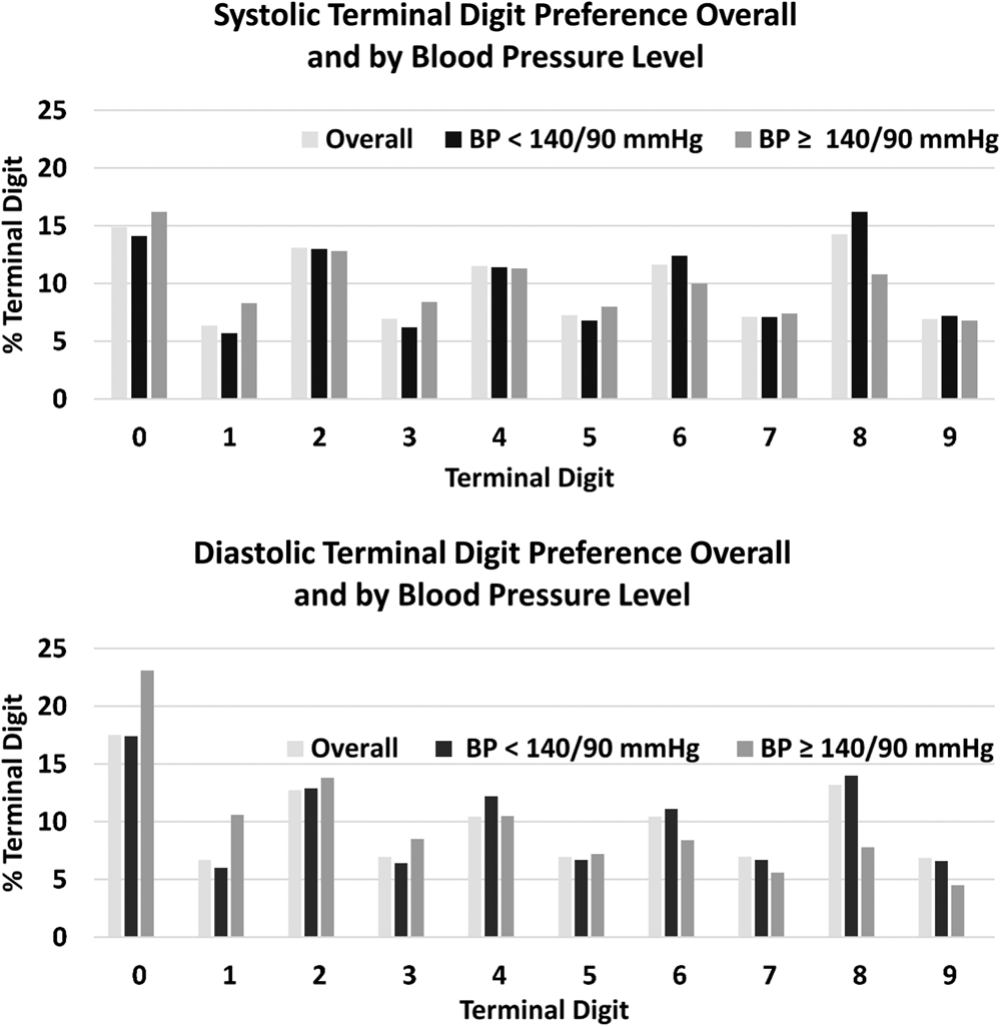
Terminal digit frequency for systolic (top figure) and diastolic (bottom) pressure in blood pressure measurements from 98,433 Veterans with a hypertension diagnosis overall and by blood pressure level.

**FIGURE 3:**
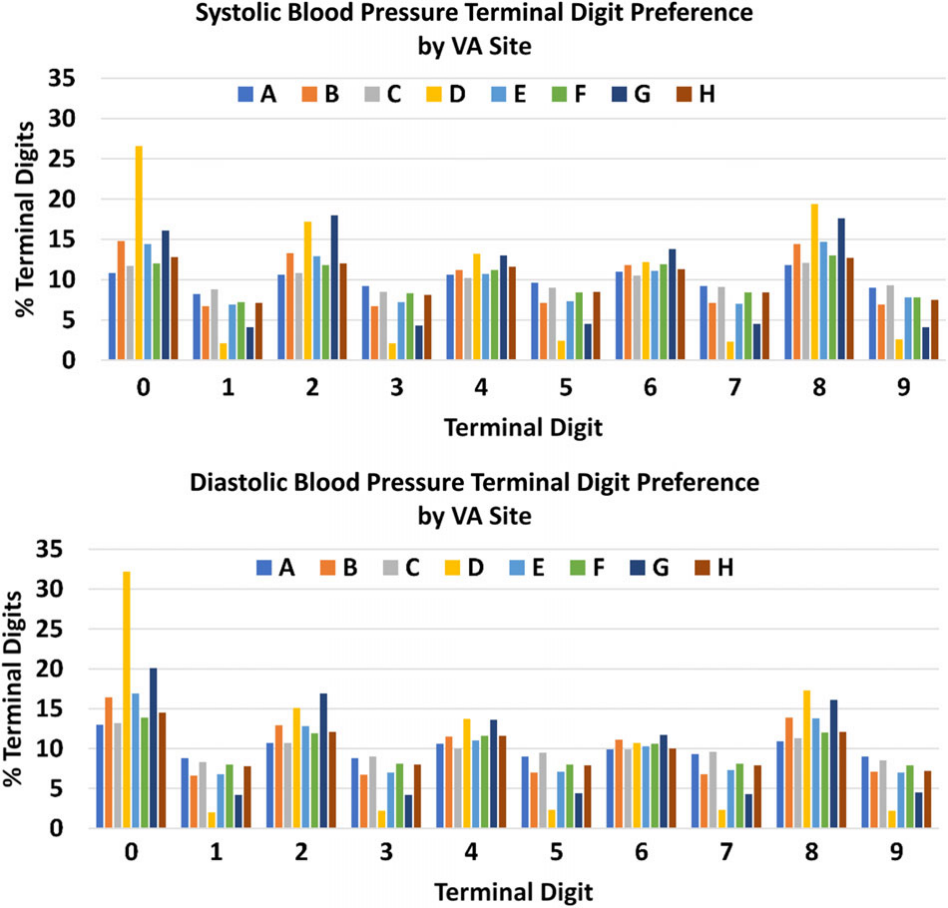
Terminal digit frequency for systolic (top) and diastolic (bottom) blood pressure measurements in 98,433 Veterans with a hypertension diagnosis by VA medical center.

Figure 2 shows the frequency of terminal digits for SBP and DBP overall and by BP level (<140/90 or ≥140/90 mmHg). The distribution of terminal digits 0 through 9 differed significantly from 10% in the pooled sample (*P* < .001) and for BP measurements < 140/90 mmHg (*n* = 489,934 BP measurements) (*P* < .001) and BP measurements ≥140/90 mmHg (*n* = 70,863 BP measurements) (*P* < .001). Digits 0 and 8 were the most common terminal digits recorded for SBP (14.9% and 14.3%, respectively) and DBP (17.5% and 13.2% respectively). For all eight centers, even numbers were preferred for terminal digits. As shown in Figure 2, terminal digit 8 was the most frequent terminal digit for SBP measurements <140/90 mmHg while terminal digit 0 was most common for SBP measurements ≥140/90 mmHg. Similar findings were noted for DBP measurements. Figure 3 shows the TDP for SBP (top) and DBP (bottom) by VA site.

**FIGURE 4:**
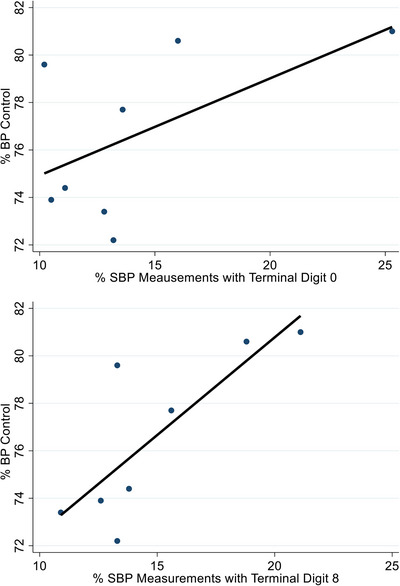
Percent blood pressure control (<140/90 mmHg) within a Veterans Affairs (VA) medical center, based on last clinic visit recorded vitals, versus frequency of terminal digit zero or eight for systolic blood pressure (top) and diastolic blood pressure (bottom) in 98, 433 Veterans with a hypertension diagnosis. Each circle represents a VA medical center.

All centers showed significant TDP for both SBP and DBP (*P* < .001 for all sites) but level of TDP for a given digit differed substantially by center. Across the 8 centers, frequency of SBP terminal digit 0 ranged from 11% to 35% for BP measurements ≥140/90 mmHg and from 10% to 24% for BP measurements <140/90 mmHg (see supplemental Figure S1). Figure 4 shows the BP control rates for each of the 8 centers with their corresponding frequency of terminal digits 0 and 8 for SBP (top) and DBP (bottom). The centers with the highest BP control rates had the highest TDP for 0 and 8.

### 3.2 Qualitative analyses

Thirteen interviews with four LPNs, three nurse managers and six physicians working in PACT clinics were completed. Table 3 provides data on respondent's perceptions of BP measurement protocols and BP targets. Most interviewees reported BP was measured in their PACT clinic using semi‐automated (Dinamap) devices and aneroid sphygmomanometers (auscultatory method); only one interviewee reported BP was measured solely with semi‐automated devices (Table 4). No interviewee reported use of automated office BP (AOBP) within their clinic. Overall, only 6 out of the 13 interviewees were aware of a clinic policy for BP measurement for their respective VA medical center.

**TABLE 3:** Interview responses on blood pressure measurement and policies at their PACT.

Interviewee site	Role	Reported method of BP measurement	BP measurement policy	BP goal to trigger additional BP measurement
D	Physician	Semi‐automated (Dinamap) and auscultatory	No	120‐125 mmHg with semi‐automated BP measurement and 125‐130 mmHg with auscultatory BP measurement for high CVD risk, all others <140/90 mmHg
D	Physician	Semi‐Automated and auscultatory	Uncertain	No set goal
D	LPN	Auscultatory (better with auscultatory than Dinamap)	Yes	No set level
D	Nurse Manager	Semi‐automated and auscultatory	Yes	<130/80 mmHg
D	Physician	Semi‐automated and auscultatory	Yes	<140/90 mmHg
F	LPN	Semi‐Automated	No	<130/80 mmHg
F	Nurse Manager	Semi‐Automated and auscultatory	Yes	<130/80 mmHg
F	Physician	Semi‐automated and auscultatory	Uncertain	<140/90 mmHg
F	Physician	Semi‐automated and auscultatory	No	<140/90 age <85 years and <150/90 age ≥85 years
H	LPN	Semi‐Automated	No	<140/90 mmHg
H	Physician	Semi‐automated (Dinamap) sometimes auscultatory	No	<140/90 mmHg
H	LPN	Semi‐automated‐ one provider requests auscultatory BP only	Yes	<140/90 mmHg
H	Nurse Manager	Dinamap and auscultatory	Yes	AHA guidelines

**TABLE 4:** Results of interviews by measure Accurately, Act rapidly, Partner with patients.

Role	Center	Measure accurately
Physician	F	“Manual BP measurement is more accurate…”
LPN	H	“One of our providers asks all LPNs for manual BP only”
Physician	H	“Use Dinamap to measure BP 90% of the time… physicians rarely measure BP”
LPN	D	“Semi‐Automated BP are prone to error….PCP will check BP manually if repeat BP high”
Nurse Manager	D	"BP readings were too high and better on manual"
Physician	D	“Notify RN or PCP via teams especially if assistance is needed for BP measurement, questions on the measure validity..”
Physician	F	“A lot of times BP is not entered into the chart. LPN lets him know to trigger him to recheck the BP”
Nurse Manager	H	“[Electronic] BP measurement devices to cycle in one‐minute intervals and calculate an average office BP reading are available in clinic but this cycling option is never utilized”
LPN	H	“I usually take at least two BP assessments”
Role	Center	Act rapidly
Physician	D	“When abnormal, teams notification from LPN patient is ready vitals are taken and will also say if BP is very high or low; if patient is late, will send to PCP for another assessment. If patient is on time/time allowing, take another BP after five minutes…”—
Role	Center	Partner with patients
LPN	H	“..blood pressure wallet card, which has little white cards with date, time, pulse, and weight. A little slot to jot down those details, add am pm or event exact time. Some of them will religiously take their BP at home, some will bring the card to their visit, we copy it and put it into their vitals”
LPN	F	“If BP is high we… look at the history and ask questions about their history. What kind of diet are they on? What kinds of stress are they under? We'll educate them, set them up with a nutritionist, give them water (if low), and we let the provider know what's going on.”
LPN	H	“I [go] .. over the BP medication, taking as prescribed, taking anything over the counter, smoking, exercising, lifestyle changes‐ anything that they mention as having changed.”
LPN	H	“Provider might take a BP when they come in, LPN writes it on her sheet. There will always be a follow up (visit) if it's (BP) very high.”
Physician	H	“During intake, PCP is made aware and it's up to PCP on what to do with patient. PCP doesn't usually take another BP. The provider would ask the PT to take a BP log over 7 days. Will issue a VA BP machine if not already given to patient and will go over how to properly use it. Patient will be asked to follow up.”
LPN	H	“Patients at VA can make hypertension appointments which specifically focus on BP management”

Table 4 shows the interview results presented by the MAP themes.^16^, ^17^, ^18^ Measure accurately depicts how centers measure BP and three of the interviewees voiced the opinion that auscultatory methods using aneroid sphygmomanometers provide more accurate BP measurements than use of semi‐automated oscillometric devices. Two interviewees from two separate centers reported that elevated BP measurements are frequently not recorded in the EMR until they are confirmed by an auscultatory BP measurement using an aneroid sphygmomanometer by the physician. While all interviewees reported that LPNs routinely conduct clinic BP measurement (Table 3), eleven out of thirteen interviewees stated that physicians perform auscultatory BP measurements with aneroid sphygmomanometer in certain situations, for example, the BP measurement is deemed too low or too high, or patient arrives late to clinic.

Act rapidly focuses on the response to an abnormal BP value. Stated BP goals by the interviewees were not consistent but most reported a BP goal <140/90 mmHg (see Table 3) for patients. Once BP is determined to be above goal, interviewees indicated that the patient's BP measurement is repeated. A high BP reading may involve escalating care by notifying a registered nurse or PCP of the abnormal value, which may include physicians performing repeat auscultatory BP measurements.

Partner with patients encapsulates efforts to work with hypertensive patients to achieve BP control. Most respondents discussed the importance of elucidating reasons for BP elevation in a given patient (Table 4). Interviewees also noted that Veterans can obtain electronic BP devices at minimal or no cost from the VA to self‐monitor home BP and schedule follow‐up nurse or PCP clinic visits to specifically address BP management.

## 4 DISCUSSION

In this study, we examined the distribution of terminal digits for both SBP and DBP in hypertensive Veterans across 8 VA medical centers. For both SBP and DBP, TDP was present in all centers but level of TDP differed by center. All 8 centers showed preference for even numbers when recording BP, which reflects use of auscultatory methods. Deciles on manometer scales are divided into 2 mm increments so even digits are usually recorded.^13^, ^21^, ^22^ Many interviewees stated that elevated BP measurements using oscillometric devices in clinic are often followed by auscultatory BP measurement using aneroid sphygmomanometers. In addition, several respondents stated a preference for the auscultatory method. These findings demonstrate that VA PACT clinics continue to rely on auscultatory methods, especially if the initial BP reading is high. Medical centers with the largest TDP for 0 and 8 had the highest BP control rates, which suggests that estimated prevalence of BP control may be influenced by TDP. We have previously reported an increase in mean clinic SBP and DBP within a single PACT after transitioning from auscultatory to automated methods for BP measurement.^14^ The lower BP readings with auscultatory methods could have in part been due to TDP leading to inflated BP control rates. Lack of calibration of aneroid sphygmomanometer devices could also influence BP measurement accuracy.^10^ Accuracy of BP measurement remains essential for hypertension management^1^, ^10^ and successful programs to improve hypertension management include standardized protocols for BP measurement with the use of validated oscillometric devices to eliminate TDP.^14^, ^23^, ^24^

The average BP over a prolonged period of time is the most important predictor of long‐term CVD events.^25^ Clinics measure BP to assess the patient's need for initiation or modification of hypertension treatment to reduce CVD risk. However, a single clinic BP measurement may not provide accurate estimates of long‐term average BP and CVD risk due to the beat‐to‐beat variability in BP.^21^ Clinic BP may also be measured with poor technique and then recorded with preference for a certain terminal digit; these sources of error will further lower the correlation of a single clinic BP measurement with a patient's average BP over an entire day.^10^, ^21^ Clinic BP measurement can be improved with use of AOBP. In the clinic setting, automated oscillometric devices can measure BP at one‐minute intervals after a five‐minute rest for a total of 3 measurements and then calculate an average BP or AOBP.^10^ Compared with auscultatory methods, AOBP in clinic settings mitigates the white coat effect, eliminates digit bias, and provides BP estimates that are closer to awake average BP measured with ambulatory BP monitoring (ABPM).^10^, ^26^ In this study, no interviewee mentioned use of AOBP. The VA DoD hypertension guideline^9^ states that AOBP with an average of 3 BP measurements is the preferred method for BP measurement while auscultatory techniques with an average of 2 or more readings may also be utilized in outpatient settings. Use of AOBP does require more time than a single BP measurement but this burden may be mitigated if AOBP is performed only when the initial BP measurement is elevated.

The Veteran population in this study involving 8 VA medical centers was older and most had high CVD risk based on age and co‐morbidities. The rate of BP control <130/80 mmHg in the Veterans with high CVD risk was less than 40%. In the general U.S. population, approximately 25% of hypertensive adults have BP controlled to <130/80 mmHg.^27^ The AHA/ACC Hypertension guideline recommends a BP goal <130/80 for hypertensive adults with known CVD or with 10‐year atherosclerotic CVD event risk ≥ 10%.^1^ The VA DoD Hypertension Clinical Practice Guideline suggests treating all adult patients to a SBP level <130 mmHg, but also emphasizes the importance of shared decision making and individualization of treatment goals.^9^ Interviews with frontline staff reflected variable and inconsistently reported or established clinic BP goals. Efforts are needed to educate both Veterans and clinicians on the benefits and risks of a BP goal <130/80 mmHg for adults with hypertension.

The study features several strengths, including the large sample size and inclusion of data from multiple clinics within 8 VA medical centers located throughout three U.S. states. The reported BP control rates and prevalence of TDP in this study may not be generalizable to other VA medical centers across the U.S. Data were pulled by VA medical center and we were not able to discern BP control or TDP by specific PACT clinic. Due to the restrictions on the scope of research, interviews were only conducted at 3 of the 8 VA medical centers. Our analyses focus on TDP and do not assess other important aspects of BP measurement such as proper positioning of patient, proper cuff size and appropriate rest period prior to BP measurement. In addition, our analyses did not address use and methods of home BP monitoring and the results of these interviews may not reflect the views and/or practice patterns of PCPs at other VA medical centers’ primary care or subspecialty clinics.

In summary, our analyses show significant TDP across 8 Midwest regional VA medical centers. Interviews with PACT clinicians suggest low use of AOBP and continued use of auscultatory BP measurement, especially to confirm an elevated BP measurement. While approximately 70% of Veterans with diagnosed hypertension in this study had BP control defined as <140/90 mmHg, only about 40% of those with high CVD risk had a BP <130/80 mmHg. Future studies should examine strategies for improving BP measurement to reduce TDP and evaluate interventions that improve BP control, especially in Veterans with high CVD risk.

## CONFLICT OF INTEREST STATEMENT

Dr. Kramer reports receiving consulting fees from Bayer Pharmaceuticals. No other author reports any competing interests.

## Supporting information

Supplementry Information

## Data Availability

Data are available to researchers with VA Institutional Review Board approval through an application process through the VA Corporate Data Warehouse.
