# Supplementary material for: Blood pressure measurement and blood pressure control in Veterans Affairs medical centers
Source: J Clin Hypertens (Greenwich). 2023 Jun 21;25(7):601–9. doi: 10.1111/jch.14684 (PMC10339365; doi:10.1111/jch.14684)

Supplemental Figure 1. Terminal digit frequency for systolic (top) and diastolic (bottom) blood pressure measurements in 98, 433 Veterans with a hypertension diagnosis by VA medical center. Terminal digit frequency shown for blood pressure (BP) measurements < 140/90 mmHg (upper and bottom left panels) and ≥ 140/90 mmHg

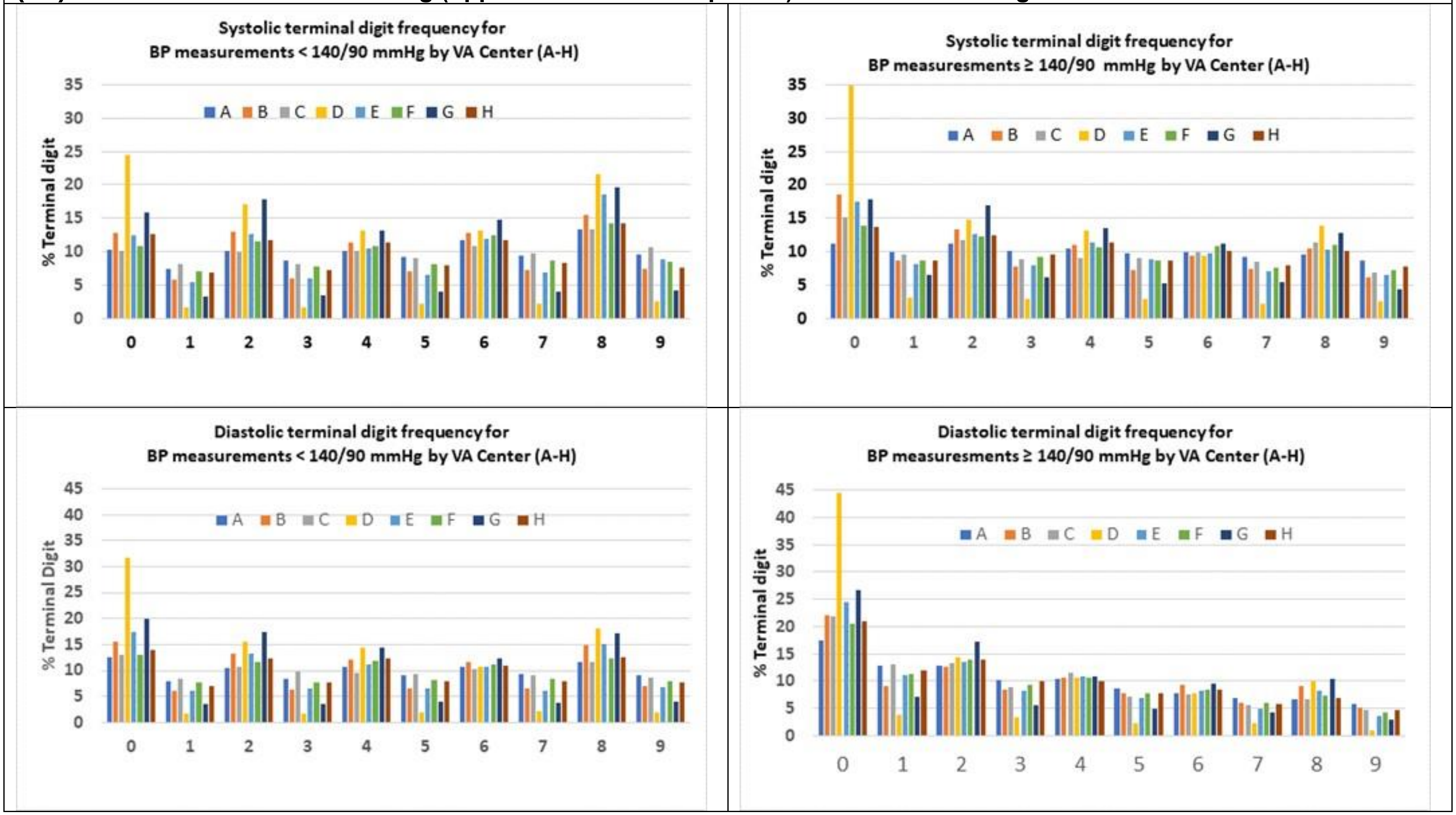

Supplement: Supplementary file 1 — Supplementry Information [file JCH-25-601-s001.pdf]
